# From Bowditch to beta-blockers: evolution of the understanding of the importance of heart rate and myocardial energetics in cardiomyopathy

**Published:** 2009-02

**Authors:** James Ker

**Affiliations:** Department of Physiology, University of Pretoria, Pretoria

## Abstract

**Summary:**

During the past three decades, every aspect of cardiomyopathy has undergone dramatic change. When examining the literature on the physiological aspects of the failing heart, one immediately recognises that South Africa has made a contribution: Brink, Bester and Lochner evaluated the possible therapeutic aspects of the Bowditch phenomenon and myocardial energetics in cardiomyopathy almost four decades ago, at a time when the condition even had another name, myocardiopathy.

## Summary

All aspects of cardiomyopathy – from our knowledge on ultra-structural and physiological alterations, to pharmacological approaches to therapy, surgical treatment modalities and later device-based therapies – have undergone dramatic changes during the last three decades. Even the terminology has changed. If one scrutinises articles from the 1950s to the 1970s one will find that the preferred term then was ‘myocardiopathy’.

When analysing the progression of knowledge on the physiology of the failing heart that has made an impact on therapeutic advances over the past 30 years, I am proud to state that South Africa has made a contribution. In 1972, the following article by Brink, Bester and Lochner appeared:^1^ A comparison of stimulation frequency and electro-augmentation on myocardial function, extensibility, coronary flow rate, oxygen consumption and glucose metabolism.

Thirty years later, we would see dramatic paradigm shifts regarding the importance of heart rate in cardiomyopathy. Today we understand the importance of heart rate variability and heart rate turbulence as prognostic markers in various cardiovascular disorders and we have conclusive evidence from clinical trials that reducing heart rate in cardiomyopathy confers a survival advantage.

## The Bowditch phenomenon

When cardiac myocytes are stimulated at faster rates, they increase their force of contraction.^2^ This ability of the vertebrate heart is central to survival and is known as the Bowditch phenomenon. 2,3 It is also known as the ‘treppe’ or staircase phenomenon.

Henry Pickering Bowditch, famed physiologist (nephew of the well-known Boston physician Henry Ingersoll Bowditch) and later dean of Harvard Medical School,^4^ published his classic article in 1871, describing the positive inotropic response of the heart when the heart rate increases. The next 100 years would see many articles examining the response of the myocardium to various stimulation frequencies, effected by electrical devices external to the heart.^2^

It would be many years after Bowditch’s article before it became apparent that the failing heart behaves very differently to an increase in heart rate. The failing heart does not exhibit a Bowditch phenomenon – there is no increase in the inotropic response to an increase in heart rate,^5^-^7^ with some failing hearts even exhibiting a reverse Bowditch response. It was during this era that the article by Brink *et al.*^1^ raised the issue that it was doubtful whether the phenomenon of increasing heart rate could be used for therapeutic purposes in the failing heart. Today we have ample clinical and laboratory evidence that reducing the heart rate improves the prognosis of patients with heart failure.

In 1967, Brink *et al.*^8^ published an article on the work performance of the isolated, perfused, beating heart in Syrian hereditary cardiomyopathic hamsters. In 2007, exactly 40 years later, work on similar Syrian cardiomyopathic hamsters clearly demonstrated that the chronic administration of carvedilol (a beta-blocker) improved cardiac function.^9^ This was in striking contrast to the line of thought in 1972, when the Bowditch staircase phenomenon was being explored as a possible therapeutic modality in heart failure. Already in 1972, work by Brink *et al.*^1^ had raised the question that this would not be a viable therapeutic option, thus paving the way for a major paradigm shift and the current therapeutic knowledge to use beta-blockers in heart failure patients.

## The failing heart as ‘an engine out of fuel’

Another important concept realised today in ‘modern’ cardiology is that the failing heart, as opposed to the normal heart, can be viewed as ‘an engine out of fuel’.^10^ In 1939, Herrmann and Decherd^11^ published an article on the chemical nature of heart failure. However, interest waned over the next few decades, only to be revived in the 2000s with Taegtmeyer^12^ elegantly summarising the situation as: ‘Metabolism – the lost child of cardiology’.

The human heart displays an enormous energy requirement – 6 kg of ATP every day.^10^ If this requirement is not met, it will result in the reduction of mechanical energy delivered to the actin–myosin interaction process and a drop in the contractile ability of the myocardium. However, we still do not possess an accurate method for determining the levels of ATP and phosphocreatine near the sarcoplasmic reticulum in the intact, *in vivo* human heart – they are extrapolated from global measurements using ^18^F-FDG PET imaging.^10^

Already in their 1972 article, Brink, Bester and Lochner^1^ had realised the importance of ‘myocardial energetics’,^10^ and glucose uptake and lactate production were analysed when evaluating the Bowditch phenomenon in the isolated, perfused rat heart. Unfortunately, in this case scenario, more than 30 years later we still do not possess the ideal, reliable way to measure myocardial energetics where we need to – in the peri-myofibrillar space, near the sarcoplasmic reticulum and sarcolemmal ion pumps.^10^

Therefore, I conclude that this historical article by Prof AJ Brink *et al.*^1^ was one of the bricks that paved the way to the current understanding and use of beta-blockers in patients with heart failure and, furthermore, that it should also be an inspiration to find new and better methods for measuring ‘myocardial energetics’ – cardiology’s lost child, in order to find a whole new therapeutic armamentarium to treat the ‘engine out of fuel’.
